# Supplementary material for: Streptomyces venezuelae uses secreted chitinases and a designated ABC transporter to support the competitive saprophytic catabolism of chitin
Source: PLoS Biol. 2025 Aug 6;23(8):e3003292. doi: 10.1371/journal.pbio.3003292 (PMC12370203; doi:10.1371/journal.pbio.3003292)
Supplement: Attachment — Submitted filename: Response to reviewers_final.docx [file pbio.3003292.s006.docx]

Roland Roberts, PhD
Senior Editor
PLOS Biology

Dear Dr. Roberts,

Thanks very much for your interest in this work and for supporting its review. We have revised the manuscript significantly in response to the reviewers’ comments.

Reviewer #1 requested co-culture experiments with additional bacteria to expand the cross-feeding result to other species. In fact, we had previously tried to do so with *Pseudomonas aeruginosa, E. coli, Rhodococcus jostii* and a demonstrated ‘exploiter’ bacterium that we sourced from Prof. Uwe Sauer. Unfortunately, these experiments did not provide any additional insight into the cross-feeding phenomenon though, as outlined in our response to the reviewers (see below), this was for technical reasons and does not compromise the results we have reported with *Bacillus subtilis*. Importantly however, none of these other bacteria were able to grow on our chitin medium. We have added this data to the manuscript as it underscores the importance and relative rarity of the *Streptomyces* chitinolytic system that we have described. We hope this and our replies to the reviewer (see below) will be satisfactory for acceptance.

Specific responses to the reviewers’ comments can be found below. Thanks again and all the best.

Sincerely,

Justin Nodwell and Anne van der Meij

**Response to reviewers**

**Reviewer #1:
[identifies himself as Glen D'Souza]

This manuscript presents a well-executed and compelling investigation into the chitinolytic metabolism of Streptomyces venezuelae. The authors demonstrate that S. venezuelae can use raw, insoluble chitin as a sole carbon and nitrogen source, outperforming glucose in both metabolic activity and sporulation. They identify a suite of chitinases and establish the central role of the DasABC transporter in chitobiose uptake—both for metabolic competence and for limiting nutrient access to Bacillus subtilis in co-culture. The integration of transcriptomics, targeted mutagenesis, LC-MS, and microbial competition experiments strengthens the manuscript substantially. Overall, this study makes a novel and significant contribution to microbial ecology, physiology, and our understanding of nutrient competition in soil ecosystems.**

Thank you for your kind words and highlighting the contribution of our work with respect to microbial ecology/physiology and nutrient competition. We’re also grateful for the suggestions on how to further improve the manuscript.

**I have some points where the manuscript can be improved further.

1. The story has broad biological and ecological relevance, but the title is narrowly focused. I encourage the authors to consider a more general title, such as: Ecological Specialization in Chitin Catabolism by Streptomyces Supports Competitive Growth and Nutrient Sequestration, or A Conserved Strategy for Competitive Chitin Metabolism in Soil Bacteria**

We are very happy to adjust the title to a more general one. Another reviewer pointed this out as well. Thank you for the suggestions! We’re now proposing “Competitive saprophytic metabolism of chitin by terrestrial bacteria”

**2. The authors convincingly show that DasABC restricts chitobiose access to B. subtilis, but testing an additional soil microbe (e.g., Pseudomonas fluorescens) in co-culture would substantially broaden the ecological implications and reinforce the generality of nutrient privatization.**

We agree that broadening this effect to additional species of soil microbes would strengthen the case and have made serious efforts to do so (much of this work predated the initial submission). We first tried to use *E. coli, Pseudomonas aeruginosa* and *Rhodococcus jostii*. What we observed with these is that, in defined chitin medium, all three were completely overgrown by *Streptomyces venezuelae*. This is not surprising as, aside from the chitin, this medium is extremely nutrient poor and none of these bacteria encodes an obvious chitinolytic system. When repeated in the presence of the *dasBC* mutant, none of the three experimental ‘exploiters’ grew efficiently. We interpret this to mean that none of them can import chitobiose or any other catabolites efficiently enough to support growth.

Furthermore, we also acquired a demonstrated exploiter species SilA3R06 of the marine genus *Silicibacter* from Uwe Sauer (Pontrelli et al., 2022, Science 
DOI: 10.1126/sciadv.abk3076). Co-culture with this bacterium proved impossible because it only grows under high salinity conditions that proved too salty for *Streptomyces venezuelae* including in particular the *dasBC* mutant.

For these reasons, we are unable to provide this additional data as requested. On reflection however, we have added the negative growth data as it highlights the fact that full chitin catabolism is a rare functional capacity (Figure S 5B). Moreover, we point out that *Bacillus subtilis*, the basis of the experiment we have presented, is also a soil bacterium that is present in ecosystems around the world. We hope the reviewers will agree that this is sufficient for the case we are trying to make.

**3. Some chitinases showed no growth phenotype when deleted. A short discussion on whether this reflects functional redundancy, context-dependent activity (e.g., biofilm-associated, substrate-dependent), or differential regulation would be valuable.**

Agreed. We now included some further context in the discussion to reflect these nuances. See line 570 of the revised manuscript: “Some chitinase mutations did not confer a growth defect on chitin. Most likely these chitinases have redundant roles with other enzymes or, conceivably, serve different functions, like antifungal activity” **4. The authors should clarify whether the B. subtilis strain used encodes known chitobiose transporters. This would help contextualize its ability to exploit the extracellular chitobiose in dasBC mutant co-cultures.**

Good point. *B. subtilis* is known produce chitinases that can degrade N-acetyl glucosamine polymers into smaller oligosaccharides, including GlcNAc, but there is no direct evidence from the available literature that it can import chitobiose nor that it encodes a specific chitobiose transporter. We will address this in the discussion. See line 603 of the revised manuscript: “Importantly, while *B. subtilis* encodes chitinase-like enzymes, there is no direct evidence from the available literature that it can import chitobiose or that it encodes a specific chitobiose transporter (76, 77). ”  **5. The RNA-seq data analysis is informative, but the link between differential gene expression and the observed physiological outcomes could be drawn more clearly. For instance, discussion of regulatory patterns, carbon utilization pathways, or broader stress responses would enrich the interpretation.**

We agree that more physical outcomes could have been linked to the RNA-seq data, and we will explore some of these in future work. However, given the large number of differentially expressed genes, it is challenging to pinpoint the most relevant stress responses or regulatory pathways—particularly in *Streptomyces*, which are known for their complex regulatory networks. For this reason, we chose to focus on genes with the lowest false discovery rates and/or those supported by additional experimental evidence. **6. The manuscript infers that DasABC is specific for chitobiose. However, given the variety of oligosaccharides released during chitin degradation, please clarify whether the transporter might import longer oligomers, and to what extent its selectivity has been tested or inferred from the data.**

Thank you for pointing this out. We have indeed not tested the specificity for chitobiose of the dasABC transporter. However, when comparing spent media samples from WT S. venezuelae bacteria versus the dasBC mutant, the only catabolite that accumulated to appreciable levels was chitobiose. We have rephrased our conclusions carefully, e.g.:

**“**it (the dasABC importer) imports key products of saprophytic chitin degradation, since mutants with an impaired transporter system are severely affected in growth.**”**

**“**we found that DasABC prevented the accumulation of chitobiose into the environment during extracellular chitin catabolism,**”**

**“**We would note, however, that we have not ruled out the possibility that other compounds released by the *dasBC* mutant during growth on chitin (including other GlcNAc oligomers) contribute to the growth of *B. subtilis*.**”

7. The study highlights the role of ABC transporters in nutrient privatization. A short speculative note on how such transporters may contribute to spatial structuring or stability of microbial communities would place the findings in a broader ecological context.**

This is a great suggestion. We added the following “Also, the physical structure of soil and its organic particles limits diffusion. Therefore, transporter-mediated privatization could reinforce spatial heterogeneity in nutrient availability and promote the formation of microcolonies enriched in saprophytic species like *Streptomyces*.” See line 620 **8. The manuscript notes that insect exoskeletons contain proteins and carbohydrates in addition to chitin. It would help to briefly comment on whether these components are likely to influence growth outcomes or if their contribution is negligible compared to chitin.**

This is a valid point. We have added a comment noting that additional proteins and carbohydrates can indeed serve as carbon and nitrogen sources for Streptomyces. See line 323 of the revised manuscript: “The grasshopper exoskeleton is composed primarily of chitin in addition to proteins and other carbohydrates (62), which are all potential sources of carbon and nitrogen to *Streptomyces*.” **Reviewer #2:

Saprophytic processes play a key role in global carbon and nitrogen cycles. In this manuscript, the authors studied the chitinolytic capacities of Streptomyces venezuelae. Chitin utilization by Streptomyces species was previously shown, but typically focused on colloidal chitin and frequently in the presence of other C- or N-sources.

In this study, the authors demonstrate growth of S. venezuelae and further Streptomyces species on raw chitin (grasshoppers) and showed an even higher „metabolic activity" on chitin than compared to MYM medium. Transcriptome analysis revelead the upregulation of several chitinases and mutational analysis confirmed the relevance of several chitinases for chitin consumption. They furthermore highlight the relevance of the DasABC transporter, which was previously described as a chitobiose importer in S. coelicolor. Intriguingly, they show that the activity of this importer enables S. venezuelae to grow competitively on chitin in the presence of bacterial competitors such as Bacillus subtilis. A deletion of dasABC led to the accumulation of chitobiose in the culture supernatant and supported growth of B. subtilis.
Overall, this study offers significant insights into the molecular mechanisms underlying saprophytic lifestyles and is broadly relevant to our understanding of microbial ecology and nutrient cycling. I have only minor comments for revision of this well-written manuscript.**

Thank you for acknowledging the relevance of the work presented, as well as providing detailed pointers to improve the quality of the manuscript.

**1)      L. 60-68: the authors introduce the definition of „cheaters" and „exploiters", but the difference is not really clear to me. Also in siderophore-mediated interactions both terms exist. While a cheater is a mutant derived from a producer which lost the capability of producing the siderophore, but can still utilize it, an exploiter is specialized on the utilization of xenosiderophores. However, since both terms are not really used in the following manuscript, it only needs a brief introduction into nutrient competition and privatization strategies.**

Thank you for pointing this out! We have removed the term cheater, as it does not accurately reflect the behavior we are describing and may cause confusion. We use the term exploiter as defined in the article we reference and have included an explanation to clarify its meaning. See line 58 of the revised manuscript: “These iron-binding probes are a valuable “public good” (31), and consequently, siderophore piracy is common; competing microbes can import siderophores (and their associated iron) that had been generated by their neighbours (29). It has been suggested that “private” siderophores, which can only be imported by their producers, can be important in competitive environments (32). Another form of resource competition involves "exploiters" - microbes that rely on polymer breakdown products generated by the enzymatic activity of other organisms rather than producing these breakdown products themselves (33).”

**2)      L. 341-347; Fig. S5: Can B. subtilis grow on the minimal (chitin) medium in the presence of glucose? To exclude lack of relevant trace elements.**

Yes, B. subtilis can grow on glucose (when supplemented with a nitrogen source), but its growth is reduced compared to growth in rich medium. However, what we aim to emphasize is that Streptomyces has access to all the nutrients it needs when grown on chitin, while a bacterium like B. subtilis does not. In that sense, whether B. subtilis can grow on glucose is beside the point.

**3)      L. 366-372, please indicate whether cultures were grown on plates or in liquid cultures for transcriptomics.**

Thanks for this suggestion – we have clarified this passage.

**4)      L.402-413, the authors discuss the different chitinases encoded by S. venezuelae. I do find this information quite critical for the manuscript and would suggest to include Figure S6 in the main text.**

Thank you for pointing this out! The part of figure S6 that showed the architecture of the *S. venezuelae* chitinases is now part of figure 2.

**5)      Overall, Figures can be produced with a bit more care (e.g. harmonization of font sizes and styles, etc.)**

Thank you for pointing this out – we have adjusted them accordingly.

**6)      Figure 1: The finding that S. venezuelae shows better growth on chitin than on glucose is quite intriguing. While I do understand that simple quantification via OD is not possible, I would suggest to include CFUs time course measurements to support the conclusions based on the resazurin measurement (Fig 1G).**

This is a valuable point, and we appreciate the opportunity to clarify our approach. While OD measurements are indeed not feasible in this context, a CFU time course also does not resolve the issue. When the bacteria grow on chitin, they attach to the flakes, forming dense clusters that may contain millions of cells but yield only a few CFUs per cluster. In contrast, on glucose, S. venezuelae—a species known to fragment under standard lab conditions—tends to disperse more, resulting in higher CFU counts that still do not accurately reflect the number of bacteria in the culture. To address this discrepancy, we note that after six days of growth on chitin, the bacteria reliably sporulate, whereas this is not the case on glucose. We will therefore include a bar graph showing CFUs after six days of growth on both carbon sources as an inset in Figure 1G and elaborate on these results. See line 361 of the revised manuscript: “Furthermore, growth on the defined glucose medium did not support efficient spore formation. This is supported by CFU measurements after six days of growth: chitin-grown cultures yield approximately 10⁸ spores per milliliter, whereas glucose-grown cultures reach only 10⁶ to 10⁷ CFUs per milliliter (Figure 1G, insert).”

**7)      Figure 2: please include the information to all COG categories shown in the Figure.**

We have added this information.

**8)      Figure 3: Please include significance tests for Fig 3A. The Streptomyces CFUs represent the number of mycelial pellets. Are those similar in size for the different mutants?**

Thank you for your suggestion. In our study, each experimental condition was based on three biological replicates (n = 3) per mutant. This choice was intentional, as we aimed to compare all mutants and the wild type side-by-side in the same experiment under strictly identical conditions—including media and timing—which would not have been feasible with larger replicate numbers due to practical constraints. Given these sample sizes, we believe formal significance testing would not yield meaningful results. Instead, we report mean ± standard deviation to transparently reflect the observed variability. Importantly, we repeated the experiment multiple times per mutant, and consistently observed the same or similar trends across runs. Regarding the interpretation of CFU counts: these primarily reflect the number of spores. Mycelial pellets contribute minimally to CFUs—despite containing billions of cells attached to a chitin flake, they typically yield only a few colonies. This is why we specifically wait to perform CFU measurements until the wild type begins to sporulate.

**9)      Figure 4: Please include growth of B. subtilis on chitibiose to support the „resource-sharing" experiments, although already shown indirectly with the dasBC KO.**

We agree that this control would provide valuable insights. However, this experiment is challenging due to financial constraints: 10 mg of chitobiose costs 535 CAD, and at least 50 mg would be required to perform the first small-scale tests. Additionally, while chitobiose is a plausible candidate, we cannot exclude the possibility that other compounds released by the dasBC mutant during growth on chitin contribute to *B. subtilis* growth.

Given these uncertainties and the high cost, we have decided not to pursue this control at this stage. Instead, we have been careful with our wording to reflect this limitation, for example:

“We would note, however, that we have not ruled out the possibility that other compounds released by the dasBC mutant during growth on chitin (including other GlcNAc oligomers) contribute to the growth of *B. subtilis*.”

**10)     Figure 4D: please include a proper x axis for the time! Y axis: please indicate the emission wavelength. I would further suggest to include B. subtilis (Fig. S5) here for direct comparison.**

Thank you for drawing our attention to these points — we’ve now corrected them. With regards to adding data from S5. We’ve chosen to keep Figure S5 separate to maintain the flow of the story and to avoid complications with out-of-order figure referencing.

**Supplemental material:
-       Please include informative Figure legends for all figures, in particular Figure S1 and S2 further experimental details is needed to avoid hopping there and back between SI and main text.**

We have added this information.

**-       Figure S4: it is not really clear what is seen here. It is not „growth", but rather mycelial structures. Please include further information. It would be intriguing to see whether the resazurin assay or CFU counting assays would show the same trend for other Streptomyces species.**

To address this, we changed “growth” into “dense mycelial aggregates” to better communicate what is shown. Resazurin assays across Streptomyces species would indeed be very interesting but the interpretation is challenged by inherent differences in growth rates and the release of pigments that can quench the resazurin signal. Additionally, most Streptomyces species do not sporulate in liquid culture, rendering CFU-based quantification ineffective.

**-       Figure S5, legend: "A similar increase…" ?**

Thank you – we have corrected this point.

**-       Figure S9: „spent medium"; Please indicate the time of harvesting the different spent media.**

We have added this information. **Reviewer #3:

The paper nicely demonstrates the ability of streptomycetes to utilize and grow on insoluble chitin polymers. Gene expression patterns associated with growth on chitin are reported and the chitinase genes in the model organism S. venezuelae are identified. Not unexpectedly, three of the most highly expressed chitinase genes are needed for good growth on raw chitin. Further the transporter DasABC is shown to be involved in uptake of degradation products from chitin, including chitobiose. When DasABC is inactivated, chitobiose (and perhaps other soluble products of chitin degradation) are accumulated extracellularly and can be utilized by other bacteria, as demonstrated here by allowing growth of B. subtilis.

Technically, the work and the documentation of it are great quality, and the conclusions are founded on clear and conclusive data. Regarding the overall question, it is, like the authors make clear in the introduction, no novelty that streptomycetes often like to grow on chitin (at least the colloidal from) and have chitinases. However, this paper adds a comprehensive investigation of the growth on raw, insoluble chitin, and a test of the roles of specific chitinases and the DasABC uptake system. It provides also insight into the significance of uptake systems in the life-style of saprophytic microbes in general and their ability to compete in the environment.**

Thank you for your kind comments on the technical quality of our work and for highlighting its contribution to a broader understanding of chitin degradation and the saprophytic lifestyle of microbes. **I have only a few minor comments and suggestions.

1. The effects of mutations on growth in chitin medium were estimated by assaying numbers of cfu in cultures after a certain time. This method has its limitations, particularly in cultures with large degree of clumping, as clearly is the case in the medium based on chitin flakes. Another concern is that sporulation will have a very large effect on the numbers of cfu in cultures since it would release single-cell spores from the clumps. Thus, the results of the growth assays in Fig. 3A should be further clarified. Are the large differences in cfu in some chitinase mutants when compared to WT due to differences in ability to sporulate under these conditions, or do they reflect true differences in growth and production of biomass? The authors likely have more insights or perhaps data to clarify the results (like is done for the dasBC mutants in Fig. 3B).**

Thank you for this thoughtful comment, which indeed helps to clarify the interpretation of our results. The observed differences in CFU counts reflect differences in the ability to sporulate under these specific conditions. Since sporulation is typically triggered toward the end of the growth cycle as nutrients become limiting, we interpret the earlier and more robust sporulation of the WT as a reflection of more efficient growth on chitin compared to certain chitinase mutants. These mutants enter the sporulation phase later, but eventually reach similar CFU levels as they catch up. We’ve rephrased the discussion. See line 457 of the revised manuscript: “The null mutants of subfamily A chitinases vnz_24855 (which had the highest differential transcripts abundance on chitin amongst all chitinases) and vnz_12765 had impaired growth in chitin medium as measured by colony forming units (CFUs) assays after 6 days (Figure 3A). As a result of efficient sporulation, the wild type produced an average of 10⁸ CFU/mL in chitin culture at this time point, whereas the vnz_24855 and vnz_12765 null mutants yielded approximately 10⁶ CFU/mL, as sporulation in these strains had only just begun. It is important to note that the mutants are capable of reaching higher CFU levels, but require additional time to do so.”

We hope this explanation provides better context for interpreting the growth data presented in Fig. 3A. **2. Given that there are ten chitinases in this organism, is it not surprising that deletions of single chitinase genes have such large effects? One could have expected to see substantial redundancy. Or is it possible that several of the chitinases have specific roles and that many of them are required? This could be worth discussing.**

Thank you for raising this question and giving us the opportunity to communicate our findings better. Now that we have clarified the specific effects of deleting a chitinase (see answer previous comment), we added the following to our discussion (line 570): Some chitinase mutations did not confer a growth defect on chitin. Most likely these chitinases have redundant roles with other enzymes or, conceivably, serve different functions, like antifungal activity (71-73). The chitinase deletion mutants that exhibited a phenotype consistently showed delayed sporulation, indicating that the loss of a single chitinase gene can have tangible effects. Even these effects appear to be mitigated over time under laboratory conditions, likely due to functional redundancy within the system.

**3. A list of strains and plasmids is missing. Would be valuable to add to the Supplementary material.**

Agreed, see table S1. Thank you for pointing this out.

**Minor
* In legends to Fig. 1B and 1F, please mention how long time of incubation before the measurements.**

We have added this information.

*** Line 336: Why reference to Fig. 4A here? Typo?**

This is indeed confusing. We added the data we were referring to in figure 4 to figure 1 (see 1G - insert)

*** Lines 118 and 447: This is not the right reference for MYM medium.**

We have referenced the *Streptomyces* manual which is where we got the protocol for making MYM medium.

*** Legend to Fig. S4: Good to say what chitin this was or what type of growth conditions.**

We have added this information.

*** Similarly, it would help the reader if the criteria for inclusion in Table S5 were mentioned in the Table, for example as a footnote.**

We have added this information.

*** Slight rephrasing could be done in the two final sentences (lines 636-640). Streptomyces is a group of organisms, and it becomes a bit strange (and maybe not the best style) to talk about them as a single entity. ("Streptomyces achieves…"; "its evolutionary success…"; "Streptomyces plays key roles").** 

Thank you for pointing this out, we adjusted accordingly. **Reviewer #4:

Summary: van der Meij et. al. demonstrate that the model streptomycete S. venezuelae can effectively colonize insect caracasses, metabolize chitin, and compete with potential exploiters for liberated resources from extracellular chitin degradation. Importantly, they identify multiple extracellular chitin degrading enzymes and demonstrate that effective competition for liberated catabolites results from efficient uptake via the DasABC transporter. In all, the authors offer a compelling account of chitin utilization by streptomycetes, demonstrating that these organisms may play in important role in terrestrial chitin turnover. My reaction to this work is overwhelmingly  positive; I have only one major concern (which is not even that major), and a few minor concerns. Most of my minor concerns revolve around term usage, which I think is a little loose throughout the manuscript. Beyond this, the paper is very well written and I quite enjoyed reading it.**

Thank you for your generous and thoughtful evaluation of our work. We’re grateful for your positive remarks on the manuscript’s contribution and we appreciate your helpful suggestions. **Major concerns: My only major concern is with the title, which starts with the phrase "A metabolic pathway". This sets the expectation the paper will offer a new metabolic pathway for chitin metabolism. There are no pathways in this paper. I might suggest that the title could simply be: "Competitive saprophytic metabolism of chitin by terrestrial bacteria"**

We love the proposed title! Thank you for the suggestion!  **Minor concerns:

L12: Why is the N and C in chitin considered fixed? The organisms with exoskeletons are fixing neither. In this sense, chitin is like any other C/N source.**

We've removed “fixed”.  **L14: Chitin is indeed found in exoskeletons, but what about fungal biomass? My feeling is that chitin in fungal biomass probably far exceeds the amount found in arthropod exoskeletons.**

Fair point. We do mention insects and crustaceans as non-exclusive examples, and we also refer to the fungal cell wall in line 70. **L60: "cheaters". I am taking issue with 'cheaters' here. I suggest that the word cheater implies that that they were once a cooperator that is now defecting, either through mutation or behavior. In the case of siderophore piracy, there is no prior expectation of cooperation. Almost all bacteria have uptake systems for siderophores that they cannot make. Are they all cheaters? I do not believe so. I think that the word 'pirate' or 'exploiter' is more appropriate here. This restrictive definition of cheating can be seen in the review:

Michael E. Hibbing, Clay Fuqua, Matthew R. Parsek & S. Brook Peterson. Bacterial competition: surviving and thriving in the microbial jungle. Nature Reviews Microbiology volume 8, pages15-25 (2010).

The authors cite a review (29), which does use 'cheating' to encompass siderophore piracy. However, I would suggest that this an example of 'semantic bleaching', which is the gradual weakening or reduction of a word's meaning over time, wherein the word becomes less specific and more general.**

Thank you for pointing this out and helping us improve our terminology. We have removed the term “cheater” from the manuscript, as we are not specifically describing cheating behavior, nor do we present data that supports the correct interpretation. See line 58 of the revised manuscript: “These iron-binding probes are a valuable “public good” (31), and consequently, siderophore piracy is common; competing microbes can import siderophores (and their associated iron) that had been generated by their neighbours (29). It has been suggested that “private” siderophores, which can only be imported by their producers, can be important in competitive environments (32). Another form of resource competition involves "exploiters" - microbes that rely on polymer breakdown products generated by the enzymatic activity of other organisms rather than producing these breakdown products themselves (33).”

**L63: "cheater" again. I don't think 'cheaters' here is correct since there is no expectation or history of prior cooperation. The authors correctly label them 'exploiters' in L64. I think the authors should roll with that.**

Agreed with thanks **L73: 'fixed C and N': again, aren't all biological polymers made from fixed C and N? I think none of the organisms that produce chitin fix C or N directly.**

Good point. We’ve removed “fixed” **L336: Fig 1E: Maybe consider adding an arrow that points at the green spore pellet? To a naive reader, it's not that obvious. Also, since there is no comparison here, it's kind of difficult to say what this means.**

We have corrected this as suggested.
 **L363: In what sense is chitin chemically inert? Noble gasses are inert since they do not participate in chemical reactions. I do not think the same is true of chitin. Certainly, as the authors note, chitin is insoluble and challenging for most microbes to metabolize.**

We removed “inert” from the sentence because indeed it is technically incorrect, thank you! What we meant is that it is not easily useful for metabolic purposes.  **L479: Does this mean that there were no CFUs for the das mutants, as would be assessed in 4A? Do the CFU assays only track spore count, or vegetative growth as well? If so, it should be clarified that the CFU assays only assess spore production. Seems like we are limited to the relatively subjective microscopic inspection for the most important set of mutants here. Not suggesting more experimental work should be done, just asking for clarification.**

We thank the reviewer for this careful comment. Indeed, the assays used in this study track spore counts, and we have clarified this more explicitly in the revised text. Regarding the dasBC knockout: sporulation does not occur in this mutant within the time-frame of the experiment, and CFU-based quantification is therefore not a reliable indicator for this strain (thousands of cells forming a pellet will only give rise to a couple of CFU’s at most). As a result, CFU values for the dasBC mutant are relatively low (10³–10⁴ CFU/mL). However, they present a misleading measurement, given that the quantification is technically unreliable.

**L558: Did you try the das mutants for growth on the grasshoppers? I imagine they would a have a very large defect in carcass colonization. Again, not suggesting more experimental work, but I'd love to see it!**

This is indeed an interesting idea! We have conducted some preliminary experiments however, accurate quantification remains challenging, and further optimization is needed before we can determine whether there is any measurable effect. Additionally, grasshoppers contain many other accessible nutrients, which the mutant can still utilize. Therefore, a pronounced phenotype is not a given. We intend to explore the degradation of insect exoskeleton in future work but, as this paper is foundational, have focused on the shrimp chitin, which is more highly purified.  **L584: "efficient uptake of chitin-derived oligomers limits their availability to other microbes." I would suggest that rapid uptake of liberated resources would be subject to selection. Ie. if I am going to spend resources on extracellular degradation, my uptake systems NEED to be very fast to compete against exploiters. Otherwise, this strategy will not work since I would simply lose out to said exploiters.**

We have kept the original wording in this passage however, a few lines down, we added the sentences “Also, the physical structure of soil and its organic particles limits diffusion (82, 83). Therefore, transporter-mediated privatization could reinforce spatial heterogeneity in nutrient availability and promote the formation of microcolonies enriched in saprophytic species like *Streptomyces* (83, 84).”

Hopefully, this brings the spirit of our discussion into alignment with the reviewer’s comment. **L593: No observed role for specialized metabolism. This is a bit tricky. My feeling is that 24 hours of growth is probably too short to see the effect of antibiotics, since they would typically be made later. But, then again, the authors did see robust sporulation. My point is that if the experiment went on longer, an effect of specialized metabolism might have been seen. The connection between GlcNAc metabolism and specialized metabolism alluded to by the authors in line 575 is a further hint that a connection to overall chitin metabolism might be observed in longer trials.**

This is an excellent point. It is definitely true that most *Streptomyces*  species need more time to accumulate secondary metabolites. However, we selected this timepoint because the co-culture growth curves were also performed over this duration (the ones that show that *Bacillus* doesn’t grow when WT *S.ven* is present, but does with the *S.ven* dasBC knockout). Hence, we chose our wording carefully.

The GlcNac part has an extra layer: GlcNac was found to either induce or suppress antibiotic production in *S. coelicolor*, depending on the growth medium. Thus, the link is certainly present, but the direction of the relationship is not always clear.

**L605: "Vibrio's". I'm not sure if this needs an apostrophe. I think the authors might mean 'many organisms of the genus Vibrio'.**

We have corrected this typo. **L640: "impacting everything from soil nutrient content to climate." I think this line would be more impactful if the authors provided some idea about the amount of C and N flux through chitin, if those estimates exist.**

We have not found sufficiently robust or consistent estimates. There are many unknowns, and the available rates often lack connection to other relevant data, making them difficult to interpret. We prefer to avoid excessive speculation at this point – we are not experts in this subject and, while we think that the work we have reported is relevant to climate discussions, we feel that some of that commentary is better left to true experts.
